# Do Invasive Jorō Spiders (*Trichonephila clavata*) from Asia Avoid Eating Unpalatable Monarch Butterflies (*Danaus plexippus*) in North America?

**DOI:** 10.3390/insects15050309

**Published:** 2024-04-25

**Authors:** Andrew K. Davis, Wilson Townsend Morris, Emma Hobbs, Ella Blakely

**Affiliations:** Odum School of Ecology, University of Georgia, Athens, GA 30602, USAemma.hobbs@uga.edu (E.H.); ella.blakely@uga.edu (E.B.)

**Keywords:** monarch butterflies, *Danaus plexippus*, jorō spiders, *Trichonephila clavata*, predation, palatability

## Abstract

**Simple Summary:**

Orb-weaving spiders can learn to avoid eating unpalatable prey, but what happens when they encounter one that they have never seen before? In the United States, a spider from East Asia has become established (the “jorō spider”) in recent years, and their webs are most prominent during the late-summer and fall, which is also when migrating monarch butterflies are in abundance. Since monarch butterflies are unpalatable (because of developing on toxic milkweeds), we wondered if jorō spiders would recognize this and avoid eating them, given that these species do not coexist in East Asia. Using field observations of butterflies deposited into jorō spider webs, we found that these spiders do avoid eating monarchs, and even remove them from their webs in some cases. They readily attack other butterflies, even those that are similarly colored to monarchs. This raises many questions about how they perceive the “distastefulness” of monarchs, even without tasting the butterflies first.

**Abstract:**

An invasive spider from East Asia has established in the U.S. southeast (the “jorō spider,” *Trichonephila clavata*) and is rapidly expanding its range. Studies assessing the impact of this species are needed, including how expansive its diet is. An open question is whether monarch butterflies, *Danaus plexippus*, are a potential prey item for this spider, given that jorō spiders do not coexist with monarchs in their native range. Since monarch larvae feed on milkweed, they sequester cardiac glycosides into their adult tissues, rendering them unpalatable to many predators. At sites within northeast Georgia, we staged a series of trials (n = 61) where we tossed monarchs into jorō spider webs and, for comparison, performed similar trials with another aposematic species, gulf fritillary (*Agraulis vanilla*), and a palatable species, tiger swallowtail (*Papilio glaucus*). We recorded the outcome of the trials, which included whether the spider attacked or did not attack the prey. We also conducted a visual survey during the same fall season to look for evidence of jorō spiders consuming monarchs naturally. Our findings revealed that jorō spiders avoided eating monarchs; spiders only attacked monarchs 20% of the time, which was significantly less than the attack rates of similarly sized or larger butterflies: 86% for gulf fritillaries and 58% for tiger swallowtails. Some jorō spiders even removed monarchs from their webs. From our visual surveys of the surrounding area, we found no evidence of natural monarch consumption and, in general, butterflies made up only a fraction of the jorō spider diet. We conclude that jorō spiders appear to recognize monarch butterflies as being unpalatable, even without having a prior history with the species. This invokes questions about how these spiders can immediately recognize their unpalatability without touching the butterflies.

## 1. Introduction

Orb-weaving spiders make a living by capturing insects in their webs and are one of the most dominant insect predators in most landscapes. In general, flying insects make up the majority of captured prey items [1], though most spiders are not necessarily indiscriminate in terms of what prey they target. First, web size and web placement on the landscape have a lot to do with the types of prey a spider will capture [2,3,4,5]. In addition, spiders do not necessarily consume all of the insects that become entangled in their webs, and some show a degree of “choice” based on the prey size (i.e., energetic reward), nutritional quality, or even on the toxicity of the prey [6,7,8]. Further, spiders show the ability to learn to avoid unpalatable prey items [9,10]. All of this points to how spiders have a degree of control over their own diet.

**Figure 1:**
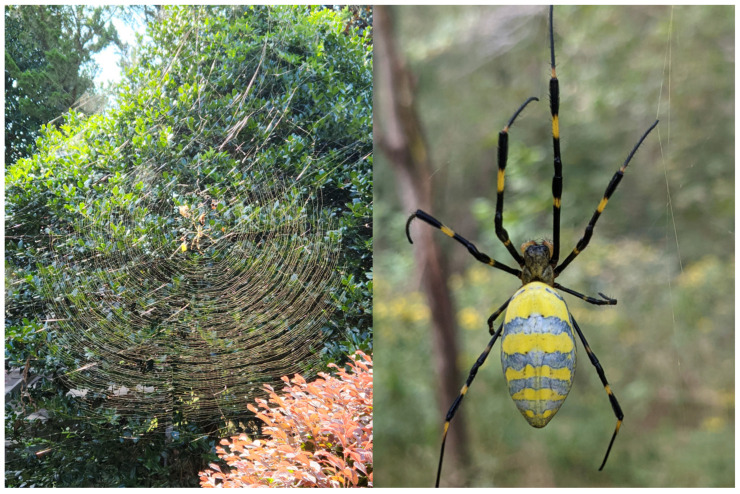
Typical web of a jorō spider, *Trichonephila clavata*, consisting of a large (1 m wide) orb, plus many intertwined support strands throughout, giving a complex, three-dimensional appearance. Right image shows a closeup of a typical mature female, which can be readily identified based on the overall body size.

In the southeastern United States, a non-native orb-weaving spider, originally from East Asia, has become established: *Trichonephila clavata*, or the “jorō spider” (Figure 1). This species was first discovered in Georgia in 2014 [11], and in the decade since, it has rapidly expanded its range in the southeast. In fact, based on the latest research that considered their physiology and climate suitability in their native range, they are expected to establish throughout much of eastern North America [12,13]. As such, there is much concern over how this new orb-weaver will impact the native fauna of this large region, including competition with native spiders for territory [13], and over their potential impact on insect populations [14]. Indeed, the large size of jorō spider webs (see Figure 1), added to their extreme density within the introduced range, means that any flying insects that frequent the same habitats and spaces will be potential prey items for this spider.

**Figure 2:**
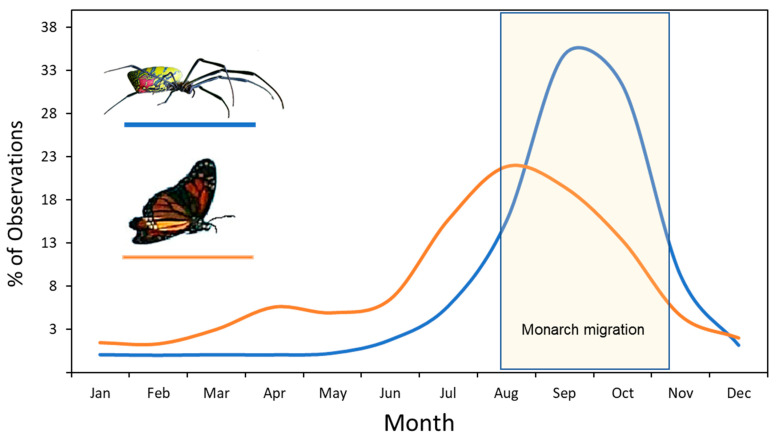
Graph showing the temporal overlap between the occurrence of jorō spiders and of monarch butterflies in North America. Both graphs were created from records downloaded from iNatualist.org, using all sightings of jorō spiders (n = 4783) and of monarch butterflies (n = 193,593) in the United States from 2018 to 2023. The approximate time period when monarchs are migrating is highlighted.

When considering potential prey species for this spider, it is important to know that jorō spider webs are the largest and most prominent in the late-summer and through the fall season. This is also the period when monarch butterflies (*Danaus plexippus*) in eastern North America are also in abundance, because they accumulate during this time for their fall migration to overwintering sites in Mexico [15]. This fall migration flyway covers most of the eastern seaboard and takes about 2 months to complete, so that waves of migrating monarchs can be seen passing through most states and Canadian provinces from mid-August to late-October (Figure 2). While migrating, monarchs require places to stop to rest each night [16] and also places with flowering plants for fuel [17]. Both of these basic needs require that monarchs must frequently fly near ground level during the journey, either engaging in feeding activities or seeking roosting sites. This could lead monarchs to become trapped in any jorō spider webs in these habitats.

It is also important to consider that monarch butterflies themselves are inherently unpalatable, as they feed on milkweeds as larvae, which contain cardiac glycosides, and which become sequestered into the adult tissues [18,19,20,21]. This trait provides adult monarchs with a degree of protection from some predators such as birds, which learn (after consuming one butterfly) to avoid subsequent monarchs [19]. This trait may also help monarchs to avoid being consumed by jorō spiders, and this is the question we addressed in the current study. What makes this question interesting is that these two species have no history of co-existing; in the native range of jorō spiders (East Asia), there are no monarch butterflies, and so we wondered if these non-native spiders would recognize monarchs as being unpalatable at all. Interestingly, there is research with related spiders in the same genus (*Trichonephila*), and with other insects that also consume milkweeds, showing how the spiders tend to reject such prey items [22,23].

**Figure 3:**
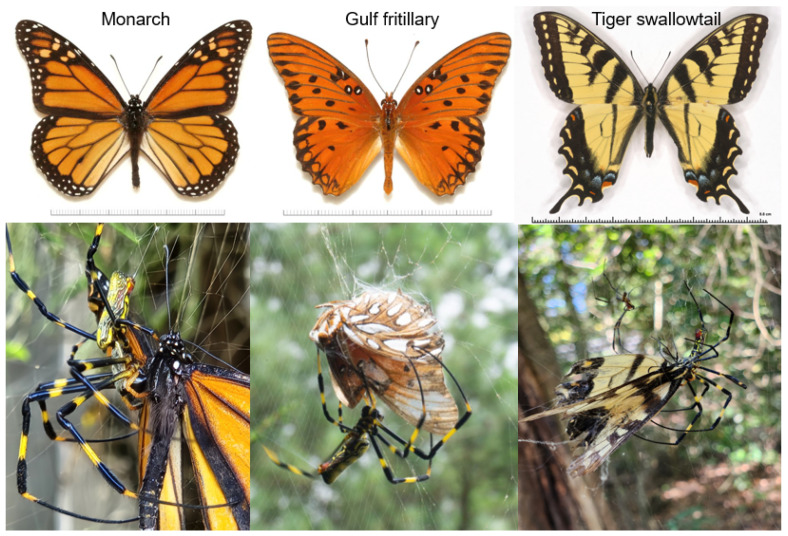
Photos of all butterfly species studied (top) and pictures of jorō spiders trapping and/or consuming each species. Top images curtesy of www.butterfliesofamerica.com, accessed on 25 March 2024. Bottom photographs by A. Davis.

As our lab is located in the introduced range of the jorō spider in North America (northern Georgia), and which is a region where monarch butterflies also travel through during each fall, this afforded us the opportunity to test this question. Thus, the goal of our study was to determine if jorō spiders show any degree of “avoidance” of monarch butterflies by directly evaluating the reactions of jorō spiders when monarchs become ensnared in their webs (i.e., tossed in the webs by us). For an interesting comparison, we also evaluated how jorō spiders react to two other butterfly species, which have different degrees of palatability or predator defense. One was the locally common gulf fritillary (*Agraulis vanilla*), which is a similar size to the monarch and also has aposematic (orange) wings (Figure 3). This species is not inherently toxic or unpalatable (i.e., its body tissue), though the adults employ emissions of adverse volatile compounds when attacked, which have been shown to deter predation by birds [24]. The other species was the yellow and black tiger swallowtail (*Papilio glaucus*, Figure 3), which is larger than monarchs though is not unpalatable [25,26]. We also conducted a follow-up, visual, survey of jorō spider webs during the same fall season and looked for any evidence of monarch butterflies being captured naturally, or, if any butterflies at all are commonly captured by this spider species. To help put the jorō spider findings into context, we conducted a small number of similar field trials involving monarchs being tossed into native garden spider (*Argiope aurantia*) webs. In addition, we searched online repositories of publicly accessible photos and recorded any observations (photographs) of native spiders capturing and/or consuming these three butterfly species.

## 2. Methods

### 2.1. Location and Spiderweb Selection

The project was conducted in Oconee and Clarke Counties, in Georgia, which is near the epicenter of the jorō spider invasion [11,27], and was carried out during September and October, when the webs are typically the largest and most obvious (Figure 2). For the butterfly trials, the authors visited properties owned by the University of Georgia, or used their own residences, and randomly selected jorō webs for testing. We only used webs occupied by a mature female that was over 2 cm in body length (including cephalothorax and abdomen), and we ensured that each web was isolated from other connecting webs (i.e., no communal webs were used). After testing, we verified that each female spider was at least this size using a ruler held next to it.

### 2.2. Butterfly Specimens

For this project, we obtained a collection of adult monarch butterflies (n = 40) from a laboratory on the university campus. These had been reared in captivity from eggs until adulthood in the lab for research purposes. As caterpillars, they had been exclusively fed cuttings of greenhouse-grown milkweed, *Asclepias incarnata*. Upon reaching adulthood, they were placed in glassine envelopes and stored at 15 °C until needed. They were fed a solution of 10% honey/water mix every 4 days during storage. For our purposes, the fact that these were captive-reared should not have been an issue, since being raised in captivity should not have affected their behavior when tangled in a spiderweb. For the comparison species, we collected adult gulf fritillaries (n = 15) and tiger swallowtails (n = 12) in the same locations as our field tests (below) using butterfly nets. Each butterfly was stored temporarily in a glassine envelope before testing with spiders (usually on the same day).

### 2.3. Spider Attack Tests

For these tests, we were interested in knowing how jorō spiders would react when one of the three butterfly species became entangled in its web. Thus, when a spiderweb was selected, one of us removed a butterfly from its envelope and held it by its wings between two fingers, and then carefully positioned themselves near the web (within 1 m), without disturbing the spider. Note that if this action did cause the spider to retreat or become disturbed in any way, we aborted the procedure and moved on to a different web. When the observer was set, they gently tossed the butterfly they were holding into the web, typically at a point midway between the web center and the edge. Since jorō spider webs are more three-dimensional than typical orb-weaver webs, this procedure had to be conducted with precision, so that the butterfly landed in the sticky portion of the central orb, and not on one of the many support strands (see Figure 1). If the butterfly did not become entrapped and/or bounced off, we considered this a failed test (and in these cases, the butterfly flew off), and we then moved to a different web. If the butterfly did become trapped, this typically resulted in the spider immediately attacking the prey item [23], and we recorded if this happened. In cases where the spider was receptive, it usually lunged at the butterfly immediately and began wrapping it with more silk, or the spider simply grabbed and held the butterfly in position while it delivered a bite (see Figure 3). If the spider did not attack the butterfly, we noted that as well. Note that either of these scenarios are readily obvious and happen usually in a matter of seconds [23]. In all cases, we waited for at least two minutes just to be sure of the outcome. For illustration purposes, we provide a video of this procedure in the Supplementary Material associated with this manuscript. The outcome of these tests was a set of observations on the frequency of attack (or no attack) for each of the three butterfly species; these data were compared between species using Chi-square tests (using the online statistical program, www.graphpad.com, accessed on 25 March 2024).

### 2.4. Monitoring the Fate of Monarchs

While conducting the butterfly trials, it became clear that the jorō spiders tended to avoid attacking the monarchs (see Results), and in many cases, the monarch was left hanging in the web, while the spider ignored it. We therefore wanted to know the ultimate fate of these trapped but uneaten butterflies, and so for a subset of the monarch trials (n = 19), one of us watched the trial from the start (i.e., when the monarch was tossed in) until it became clear what happened eventually, even if it required an hour of watching. For these cases, we recorded if the monarch was eventually attacked and eaten by the spider, whether it died in the web, whether the spider cut it out, or whether it escaped. We attempted to determine if the frequencies of “living outcomes” or “dying outcomes” differed from an even distribution using Chi-square tests.

### 2.5. Visual Survey of Prey

As a follow-up to the experimental trials above, we conducted a simple visual survey of jorō spider webs in the same area (Clarke and Oconee County, GA) in an effort to determine if any monarch butterflies (or other butterflies) are “naturally” trapped and killed by jorō spiders. This was not a systematic survey but was opportunistic in nature; one of us carried a camera for 3 weeks in September, and whenever a jorō spider was seen feeding on an insect, the author photographed the incident. This allowed us to build a photographic collection of “typical” jorō spider prey items (n = 62). From these photographs, we attempted to determine the type of prey, at least to the nearest order. By focusing only on spiders that were actively engaged in eating the prey, we could be sure the spider had definitively attacked (preferred) the prey in question. The outcome of this effort was a list of commonly consumed prey types by jorō spiders, of which we could determine how many were butterflies.

### 2.6. Trials with Native Spiders

Following the trials with jorō spiders, we conducted similar field trials involving native garden spiders, *Argiope aurantia*, which also reach maturity in the fall and have conspicuous webs during this time. In these cases, monarch specimens were tossed into the webs of the spiders as before (of mature females), and we recorded the outcome of these trials (i.e., butterfly was attacked/not attacked). A total of 9 trials were conducted, each on a different spiderweb. These trials were limited in number due to a lack of additional monarch specimens.

### 2.7. Online Observations of Native Spider Prey

To help put the jorō spider findings into context, we conducted a survey of online photographs of native spiders trapping and/or eating one of the three butterfly species. We searched publicly available photo repositories using the Google search term “spider eating butterfly”. This query pulled up approximately 720 photographs from photo archives, blogs, and news articles. We visually searched these images to screen out photos that did not actually show a butterfly or did not involve any of the three species of interest (monarchs, gulf fritillaries, tiger swallowtails). While we did not purposely screen for photos that were taken in North America, by limiting the search to only images of these three (North American) butterflies, this essentially achieved the same outcome. We then recorded the species of spider and the butterfly it was eating in the picture. This resulted in a collection of n = 33 images of 7 (native) spider species consuming these butterflies.

## 3. Results

In our field trials, we tested the reactions of jorō spiders to 35 monarch butterflies, 14 gulf fritillaries, and 12 tiger swallowtails (Table 1). As noted in the methods, no spiders were tested more than once. Jorō spiders showed a distinct avoidance of monarch butterflies; monarchs were attacked only 20% of the time, compared to 85% for gulf fritillaries (which are also orange), and 58% for tiger swallowtail butterflies (which are larger than monarchs). The frequencies of attack/no attack for monarchs were significantly different than those of gulf fritillaries (ꭓ^2^ = 18.19, *p* < 0.0001) and of tiger swallowtails (ꭓ^2^ = 6.30, *p* = 0.0122). The frequencies of gulf fritillaries and tiger swallowtails were not different (ꭓ^2^ = 2.46, *p* = 0.1166).

For a subset of the 35 monarch butterfly trials (n = 19), one of us watched the web until the outcome was clear. Out of the 19 cases, there were 10 cases (52% of this subset) where the monarch eventually escaped after struggling in the web. There were two cases where the jorō spider attempted to cut the monarch out of the web. There were six cases where the jorō spider attacked and entrapped the monarch, and then later was seen consuming it (Figure 3). One observation was ambiguous. Of the remaining 18 observations, the outcomes where monarchs were killed or died, versus those where the monarchs escaped or were cut out (6 vs. 12), were not significantly different from a 50–50 frequency (Chi-square, ꭓ^2^ = 1.03, *p* = 0.3101).

**Figure 4:**
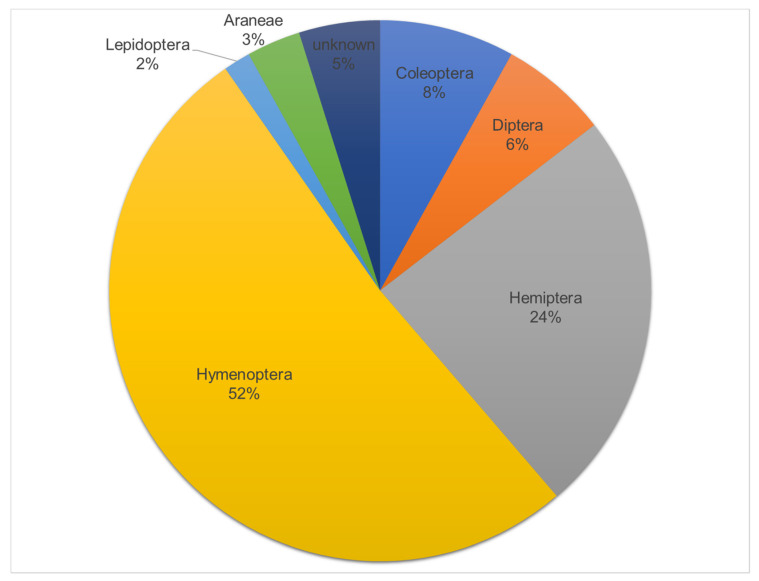
Pie chart showing the relative proportions of prey items (orders) that we witnessed jorō spiders eating during our one-month survey.

In our visual survey of “natural” jorō spider prey, we were able to photograph and identify prey types (to the nearest order) in a total of 62 different webs. In nearly all of these cases, we photographed the jorō spider directly consuming the prey. Of these, the majority of prey were insects in the Hymenoptera order (bees, wasps, and ants; Figure 4). The second-most common prey type was flies (order Diptera). We saw no cases of monarch butterflies being trapped or eaten and only found one case where a jorō had trapped a butterfly (a tiger swallowtail).

**Figure 5:**
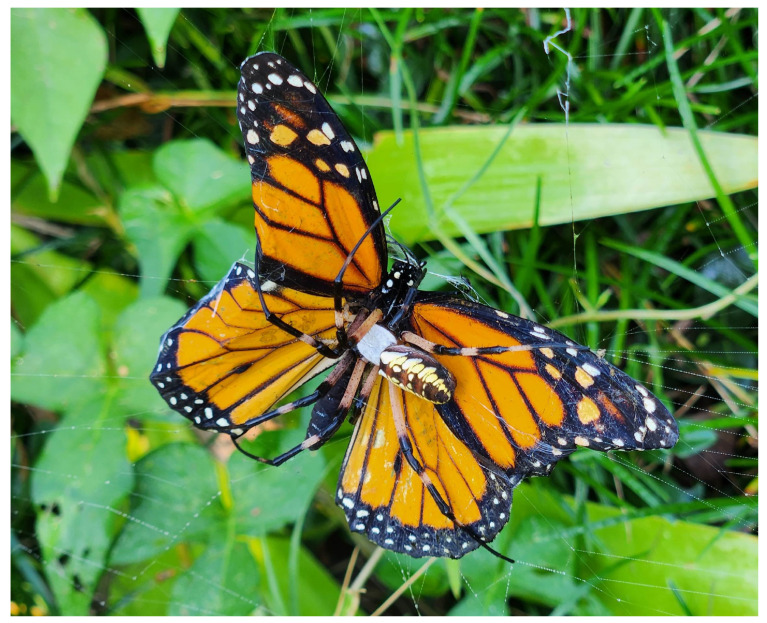
Photo of a native orb-weaver, *Argiope aurantia*, trapping a monarch butterfly that had been tossed into the web (to determine if native spiders avoid monarchs).

In the field trials involving native garden spiders (*A. aurantia*, n = 9), the spider readily attacked and subdued the monarch in seven of these trials (78%), and later, we observed the spiders eating the butterflies (Figure 5). These frequencies were significantly different than the frequencies of attacks/no attacks by jorō spiders (ꭓ^2^ = 11.02, *p* = 0.0009).

In the survey of online photographs, we found photos of seven different spider species (native to North America) that were preying upon one of the three butterfly species of interest (Table 2). We noted 19 cases where monarch butterflies were recorded being consumed by six of these spider species. Of these, 10 photographs were of garden spiders (*A. aurantia*) consuming monarchs.

## 4. Discussion

Since jorō spiders are native to a region (East Asia) where monarch butterflies do not exist, but yet their ranges in North America will eventually overlap considerably [12,13], it will be useful to know how these spiders will react when they encounter these (unpalatable) butterflies. Our research shows that jorō spiders mostly avoided eating monarchs, likely because of their cardenolide toxicity, which is a similar finding from studies of other spiders in this genus [22,23]. Meanwhile, jorō spiders do not avoid consuming the comparison butterfly species we chose, even those that are larger than monarchs (swallowtails) or those that are also aposematic (fritillaries). This finding engenders fascinating questions regarding how the spiders “know” to avoid the monarchs. Since the spiders are not native to North America, and since monarchs are not native to East Asia, we logically inferred that jorō spiders have no direct experience with monarch butterflies, yet they showed a distinct avoidance of them. Moreover, since each jorō spider only lives for one season, there is likely no opportunity for learning at the individual level (i.e., for a jorō spider to trap a monarch and learn that it is distasteful). In other words, it is unlikely that the spiders we tested had each already experienced the distaste of a monarch butterfly, especially since monarchs are typically not abundant in this region until the fall [15]. It is interesting to note that this same avoidance behavior has been found with a related spider in the same genus (*Trichonephila*), which was tested using a related milkweed butterfly in the same genus (*Danaus*), and in a completely different geographic region [23].

Interestingly, this avoidance of milkweed butterflies may not be universal among orb-weaving spiders; as a follow-up to our trials with jorō spiders, we had also tested if a native species (garden spider, *A. aurantia*) also avoided monarchs. These trials were conducted in the same manner as those with jorō spiders and using similar captive-reared monarchs. While our sample size was modest (n = 9), in seven of these trials (78%), the native spider readily attacked and subdued the monarch, and later, we observed the spiders eating the butterflies (Figure 5). Our survey of online photographs provided similar results; we found multiple records of native spiders (including 10 cases of garden spiders) trapping and/or consuming monarch butterflies (Table 2). While this simple online survey is not the same as conducting standardized field tests of preference, this evidence does suggest that at least some native North American spiders (i.e., those with experience with monarchs) are capable of consuming monarch butterflies, despite their toxicity.

In our field trials, we did note that a small percentage of monarchs were indeed attacked and, apparently, consumed by jorō spiders (n = 7, or 20%). However, it is possible that this rate is atypical, or higher than normal, because the monarchs we used had been reared on a milkweed that is naturally low in cardenolide concentrations [28]. There are over 100 species of milkweeds in North America, and each differs in their cardenolide levels [18]. There is even geographic variation in cardenolide levels within the same milkweed species [29]. All of this can translate into considerable variation in tissue toxicity (palatability) within adult monarchs. Moreover, there is also evidence that cardenolide levels can even wane over time in older monarchs [30], which could lead to even more individual variation. In the end, there may be considerable variation in palatability within wild monarchs, which could then translate into varying degrees of avoidance by jorō spiders. Perhaps future work could experimentally test if and how jorō spiders respond to monarchs that were reared on different milkweed species.

In our study, we noted that a sizeable proportion of the monarchs that were ignored by jorō spiders later freed themselves or the spiders themselves cut the monarchs loose from their web. These observations are consistent with a recent review article by Sherratt and Stefan [31], who provided evidence from multiple taxonomic groups arguing that aposematic species (like monarchs) may have evolved a degree of “capture-tolerance”, in which they are capable of being captured and handled by predators, who later decide not to eat them. Such species tend to have more robust bodies and/or wings than non-aposematic species, which presumably would be more able to withstand the predator handling. The parallels between this idea and our own findings here are noteworthy, though one wonders why the native spiders did not appear to avoid the aposematic monarchs.

Some equally interesting questions raised by this research relate to how the jorō spiders could sense (or decide) if the monarchs were unpalatable, and how they could do so within the span of milliseconds immediately after the butterfly hit the web. The speed with which orb-weaving spiders attack prey is known to be extremely fast, on the order of milliseconds [32]. Indeed, when we played back the video recordings of our trials with monarchs or other butterflies (in slow motion), we could see that most spiders would typically move to attack their preferred prey even within milliseconds of it touching the web. When monarchs were tossed into their webs, most jorō spiders typically would turn toward the prey item, initially move one body length toward the prey, but then retreat, and all within the span of one second. These observations suggest that the “decision” to not attack the prey item is also made within the span of milliseconds. Even more interesting is that the jorō spiders made these decisions without touching the monarchs in any way, nor even getting close to them. This rules out any possibility of the spiders “tasting” the butterfly before rejecting it. The fact that the gulf fritillaries (which are orange, like monarchs) were readily targeted by jorō spiders also is of interest, as this would signify that the decision is not based on the aposematic butterfly color. This observation is consistent with findings from similar prey preference trials of the related *T. clavipes*, which did not show avoidance of aposematic prey, but instead avoided those that contained cardenolides [23].

Our visual survey of natural prey items in jorō spider webs revealed that butterflies in general make up a very small fraction of the jorō diet, with the main prey base being composed of Hymenoptera and Hemiptera insects. Interestingly, these frequencies differ slightly from those of a recent study, which examined this spider’s prey base in more detail [14]. In that case, the authors had used DNA barcoding to identify prey types of jorō spiders and found that the frequencies of different types of prey differed depending on whether the gut contents were examined versus the contents of the fecal material. Based on the gut contents, the major prey items were found to be Coleoptera and Diptera species [14]. Meanwhile, the analysis of spider fecal material showed the major prey to be species of Diptera and Hymenoptera. Then, the analysis of prey items in the webs showed the major types to be Diptera and Hemiptera species. Consistent with our findings, that study did show that Lepidopteran insects made up a small fraction of the jorō diet in whichever method was used. In fact, this appears to be the case with most orb-weaving species in general; Lepidopteran prey make up less than 3% of the diet of most orb-weaving spiders, including those in the genus *Argiope* [3]. This could be related to the ability of butterflies and moths to evade capture in spiderwebs because their wing scales are designed to slough off, thereby preventing getting stuck to the web [1]. Interestingly, in our trials where we tossed butterflies into jorō spider webs, we witnessed few cases where this happened for monarchs, but multiple cases with the other two species (Davis, *unpubl. data*). This prevented us from including those specimens in the current study, but these few observations nonetheless point to some inherent species-level variation in butterfly “stickiness” that should be examined in more detail.

Another finding of interest from the recent genetic analyses of jorō spider prey by Grabarczyk et al. [14] was that the authors discovered that there was higher species richness within the killed prey taken directly from the webs than that found within the feces or gut, which suggests that the spiders do not actually consume all of the prey that becomes trapped in their webs. This could be interpreted as evidence that jorō spiders do choose what prey they actually consume. This also makes sense given certain anecdotal observations of jorō spider webs in this region, especially later in the season when the webs are the largest; one author has witnessed spiderwebs that have numerous trapped prey items throughout the web, some that are not even wrapped in silk, as if the spiders have ignored them after striking the web (Davis, *pers. obs.*). Given the impressive size of the jorō webs (sometimes 1–2 m in width), it may be that the spiders end up trapping more insects than they require, which allows them to be “choosy”. Many of these untouched prey items appear to be small, suggesting that the spiders choose more high-value items [6].

Understanding the dietary preferences of jorō spiders will ultimately help in elucidating the impact of this non-native predator on the prey base of this region. Indeed, recent evidence suggests that jorō spiders may be outcompeting native spiders for territory [13]. If this happens, it would be important to consider how the prey “preferences” of jorō spiders will modify the insect populations in those habitats, especially if the jorō spider diet differs substantially from that of native spiders, though this too is a question that deserves further study. Indeed, the rapid spread of the jorō spider in the southeast [13,27], combined with its predicted range expansion [12], means that such questions should be priorities for future study. In addition, the findings from our study highlight how much remains to be learned regarding sensory perception in these and related spiders.

## Figures and Tables

**Table 1:** Frequency of attack behaviors by jorō spiders when a butterfly was tossed into their web. Spiders were only evaluated with one prey item each (which they either attacked or did not attack), and only mature female spiders were selected for testing (see Figure 1). The frequencies observed for monarch butterflies differed from the other species which did not differ from each other (see Results).

Butterfly Species	# Attack	# No Attack	Total	Attack Rate (%)
Monarch	7	28	35	**20.0**
Gulf fritillary	12	2	14	**85.7**
Tiger swallowtail	7	5	12	**58.3**
Grand total	26	35	61	**42.6**

**Table 2:** Summary of our survey of online photographs, where we had searched for observations (photos) of native North American spiders consuming one of the three butterfly species of interest. Each column shows the number of instances (photographs) involving the spider and prey species in question.

Spider Species	Monarch	Gulf Fritillary	Tiger Swallowtail
*Araneus diadematus*	1	0	0
*Araneus marmoreus*	1	0	0
*Argiope aurantia*	10	4	3
*Misumena vatia*	0	2	1
*Misumenoides formosipes*	1	0	0
*Neoscona crucifera*	2	0	1
*Peucetia viridans*	4	3	0
Grand total	19	9	5

## Data Availability

All raw data from this study are available in the Supplementary Materials.

## Supplementary Materials

The following supporting information can be downloaded at: https://www.mdpi.com/article/10.3390/insects15050309/s1, Video S1: Demonstration of butterfly preference trials, File S1: Excel file with raw data from the study.
